# Arm Anthropometry Indices in Turkish Children and Adolescents: Changes Over a Three-Year Period

**DOI:** 10.4274/jcrpe.1574

**Published:** 2014-12-05

**Authors:** Betül Çiçek, Ahmet Öztürk, Mustafa Mümtaz Mazıcıoğlu, Selim Kurtoğlu

**Affiliations:** 1 Erciyes University Faculty of Health Sciences, Department of Nutrition and Dietetics, Kayseri, Turkey; 2 Erciyes University Faculty of Health Sciences, Department of Biostatistics and Medical Informatics, Kayseri, Turkey; 3 Erciyes University Faculty of Health Sciences, Department of Family Medicine, Kayseri, Turkey; 4 Erciyes University Faculty of Health Sciences, Department of Pediatric Endocrinology, Kayseri, Turkey

**Keywords:** adolescents, arm fat area, Mid-upper arm circumference, triceps skinfold thickness, Turkish children

## Abstract

**Objective:** Time-related changes and comparisons for mid-upper arm circumference (MUAC), triceps skinfold thickness (TSF), arm fat area (AFA) are lacking for Turkish children and adolescents. To determine the arm anthropometry indices (MUAC, TSF, AFA) in children and adolescents and to also assess the changes in these indices over a 3-year time period.

**Methods:** The data of the Anthropometry of Turkish Children Aged 0-6 Years (ATCA-06) study and the Second Study of Determination of the Anthropometric Measurements of Turkish Children and Adolescents (DAMTCA-II) were used to calculate the arm anthropometry percentiles in a total group of 6982 children and adolescents aged 28 days to 17 years. The 3rd-97th percentiles were computed by the LMS method.

**Results:** In girls, 50th percentile MUAC values linearly increased with age. In boys, 50th percentile TSF values linearly increased until 10 years of age and decreased after age 11 years, while in girls, TSF values increased linearly with age. 50th percentile values for AFA showed a linear increase in both genders with age. Significant differences were found between the 5th, 50th and 95th percentile values for MUAC and AFA obtained in the two studies (DAMTCA-II and DAMTCA-I) in both boys and girls.

**Conclusions:** The prominent finding was the significant and alarming increase in arm anthropometry indices in both genders within as short period of time as three years.

## INTRODUCTION

As is well known, body fat distribution is closely related to the occurrence and development of cardiovascular disease. Waist circumference is the most commonly used index of abdominal obesity. Recently, mid-upper arm circumference (MUAC) has been proposed as another important indicator of obesity in children. Traditionally, MUAC has been accepted to be a tool in the assessment of nutritional status (1). In 2003, de Almeida et al (2) proposed MUAC measurements as an alternative anthropometric index to monitor obesity in preschool children. This measurement has also been shown to be a useful index for obesity by Mazicioglu et al (3) in Turkish children aged 6-17 years. Fricke et al (4) demonstrated that arm muscle area (AMA) and arm fat area (AFA) correlated with fat-free mass and fat mass measured by four-site skinfold thicknesses. Age-related MUAC cut-offs have been reported for Brazilian and Turkish children (2,3). However, to the best of our knowledge, systematic and regular monitoring of MUAC is not a commonly preferred method in clinical pediatric practice.

Arm anthropometry has been used as a proxy of body composition in both clinical and epidemiological settings for decades. The cross-sectional AMA and AFA were introduced for the assessment of nutritional status of children in community settings and proposed to be better indices than direct arm anthropometry (skinfold thickness and MUAC). Including both triceps skinfold thickness (TSF) and MUAC measurements, AFA is a more extensive arm-derived index than skinfold thickness or arm circumference measurements alone (5).

The primary aim of this study was to determine the trend in the changes of arm anthropometry indices (MUAC, TSF, AFA) of Turkish children and adolescents over a three-year period. Additionally, our results may be used to compare with both local and international references.

## METHODS

**Study Design, Sampling and Participants**

The cross-sectional study sample was selected from the Central Anatolian province of Kayseri in Turkey, which has a population of more than 1.2 million. Current data were obtained from the Second Study of the Determination of Anthropometric Measurements of Turkish Children and Adolescents (DAMTCA-II), a study performed between October 2007 and April 2008 covering children aged 6 to 17 years and the Anthropometry of Turkish Children Aged 0-6 Years (ATCA-06) study conducted between September 2009 and May 2010 on 0-to-<84-month-old children. Totally, 0-to-17-year-old 6982 children and adolescents were included in this study (6,7).

Additionally, we compared our current data (DAMTCA-II) with the study of the DAMTCA-I. DAMTCA-I was a similar study performed in 2004-2005, including a total of 5553 children and adolescents aged 6 to 17 years from 17 primary and secondary schools (8,9).

Chronological age was calculated as a half year by subtracting the date of birth from the observation date (e.g. 8 indicates ages 8.00-8.99 years). The DAMTCA-II study was approved by the Institutional Review Board of Erciyes University and consent was given by the parents.

**Anthropometric Indices**

In this study, MUAC, TSF and AFA were all used to measure the adiposity of children and adolescents. All measurements were performed by well-trained health professionals. The MUAC and TSF measurements were performed twice and the mean value was recorded. All of the inter-observer correlation coefficients were ≥0.91. The test and re-test reliability of measurements were determined and coefficients of variability for the TSF and MUAC were 3% and 2%, respectively.

MUAC measurements were taken in centimeters with non-elastic tape to the nearest 0.1 mm on the left arm, halfway between the acromion process and the olecranon process. The child/adolescent stood relaxed with his/her side to the health professional performing the measurement and his/her arm hanging freely at the side; the tape was then passed around the arm at the level of the midpoint of the upper arm (8,10) and the measurement was noted.

TSF measurements were taken in duplicate on the left side of the body to the nearest 0.1 mm, halfway between the acromion and the olecranon process using a Holtain skinfold caliper (9,11).

The AMA, arm area (AA), AFA and fat percent (%) were calculated according to the following formulae:

AMA (cm^2^)=(MUAC-πTSF)^2^/4π

AA (cm^2^)=π/4 x (MUAC/π)^2^ (π =3.1416)

AFA (cm^2^)=AA - AMA, as the best indicator of body fat in school-aged children (11,12).

Fat %=AFA x 100/AA (8,13,14).

**Statistical Analysis**

In the construction of the centile curves, the LMS Chart Maker Pro version 2.3 software (The Institute of Child Health, London, UK) was used. This method is appropriate to fit smooth centile curves for the reference data (15). Box-Cox power transformations summarize percentiles for 3-month periods. To normalize the data, three quantities representing L (lambda: skewness), M (m: median) and S (sigma: coefficient of variation) were used (16). The 3rd, 5th, 10th, 25th, 50th, 75th, 85th, 90th, 95th and the 97th percentiles were computed for each gender in half-year periods. Differences between both genders in each age group were assessed by independent-samples t-test. Differences between age groups in each gender were compared with the one-way analysis of variance. Difference (comparison) between both genders in each age group were assessed by independent-samples t-test. A p value of less than 0.05 was considered statistically significant.

## RESULTS

Tables 1a and 1b demonstrate the sample size per age group for six anthropometric indices (MUAC, TSF, AMA, AA, AFA, fat %) in Turkish boys and girls. In a comparative evaluation of boys and girls in each age group, the following parameters were found to show statistically significant differences between the two genders in the indicated age groups (as shown in Table 1a): MUAC, AMA and AA in 28 d-<3-month-olds and 18-to-21-month-olds; AMA in 9-to-12-month-olds; MUAC and AMA in 12-to-15-month-olds; MUAC, AMA, AA and AFA in 15-to-18-month-olds; AMA in 21-to-24-month-olds; TSF and AMA in 2 and 8 years; TSF in 3 years; TSF and AFA in 4 and 10 years; TSF, AMA and AFA in 5, 6, 7, 11, 13, 14 and years; AMA in 9 years; MUAC, TSF, AMA, AA, AFA in 15, 16 and 17 years were (p<0.05, p<0.01, p<0.001). Tables 2-4 summarize the smoothed age- and gender-specific MUAC, TSF and AFA percentile values for Turkish children and adolescents, respectively. In girls, with increasing age, 50th percentile MUAC values linearly increased (Table 2). In boys, 50^th^ percentile TSF values linearly increased until 10 years, remained unchanged at 11 years, then decreased afterwards. In girls, TSF values increased linearly with age (Table 3). For both genders, 50^th^ percentile AFA values linearly increased with age (Table 4).

Tables 5 and 6 give the comparison of AFA and MUAC of DAMTCA-II and DAMTCA-I datasets within genders. For AFA and MUAC in boys and girls, DAMTCA-II 5th-50th and 95th percentiles were significantly different between the 28 days - <3m and 10 years age groups. DAMTCA-I 5^th^-50^th^ and 95^th^f percentiles were significantly different in the 6-to-10-year age groups (Tables 5, 6).

Figures 1 (a,b) and 2 (a,b) demonstrate the comparison of DAMTCA-II and DAMTCA-I datasets for MUAC and AFA for boys and girls. For MUAC and AFA, DAMTCA-II 5^th^-50^th^-95^th^ percentiles were found to be higher than those in DAMTCA-I for both genders through 6 to 17 years.

## DISCUSSION

This study presents the smoothed MUAC, TSF and AFA percentiles from ages 28 days to 17 years in Turkish children and adolescents and also, to the best of our knowledge, is the first study demonstrating the changes in MUAC, TSF and AFA values over a three-year time period. Comparison of the DAMTCA-II data with the DAMTCA-I data showed that the 5th, 50th and 95th percentile values for MUAC and AFA had all increased within a period of three years in both genders.

Cândido et al (17) reported AFA as the most suitable index to monitor obesity in prepubertal and pubertal boys and girls in Brazil (n=788, 6-15 years). Gultekin et al (18) have demonstrated that AFA values were higher among girls and AMA among boys, pointing out to significant sexual dysmorphism in the muscle and fat patterns. Their results also refer to the significant impact of socioeconomic status on AMA and AFA among Turkish children. Lu et al (19) found that both in boys and girls, MUAC was closely associated with BMI and waist circumference and concluded that MUAC can accurately identify overweight and obesity in Chinese children.

When using anthropometric indices such as waist circumference, health professionals should be aware that this index is affected by respiratory movements and postprandial abdominal distension. The arm anthropometry indices such as MUAC, TSF and AFA which is a derived index, are all independent of the factors mentioned above and may therefore be alternative and reliable indices for overweight and obesity. In the current study, with increasing age, linear elevations were noted in MUAC, AMA, AA and AFA values in Turkish boys and girls. TSF values on the other hand showed an increase until age 9 years and decreased afterwards. Fat % values decreased linearly from the 28 days-3 months age period to 17 years.

It is worth mentioning that within the limits of this study, it is difficult to evaluate presence of a secular trend. However, the data allow us to state that an increase had occurred in all anthropometric indices and body composition parameters related to obesity in as short a time period as three years. These trends appear to be related to gender-specific changes in fat and fat-free mass associated with puberty. Pubertal development is an important factor effecting anthropometric indices and body composition during adolescence (20). During adolescence, gender differences and age differences in fat mass, fat-free mass and regional body fat distribution become apparent. However, in the present study, it has not been possible to obtain data pertaining to pubertal staging, due to some cultural considerations. Our data also shows that while body fat increases until age 17 years in girls, it starts decreasing at age 13 years in boys.

The cross-sectional nature of the current study provides the opportunity to reflect the current situation clearly. However, differences in sample characteristics between the two studies compared may be considered as a limitation. Although sampled from the same population with simple clustering and despite the fact that the age groups are similar in both data sets, there is a possibility of dissimilarities between the two datasets. Improvement in family income may also be responsible for the significant increment occurring in a relatively short period of time (21).

In conclusion, due to the lack of international cut-off values for arm anthropometry in children and adolescents, studies aiming to produce local references may be beneficial. The prominent finding of this study was the significant and alarming increase in arm anthropometry percentile values in both genders over a short period of time. This finding requires further investigation, but points to a need for preventive interventions in order to decline obesity.

## Figures and Tables

**Table 1a:**
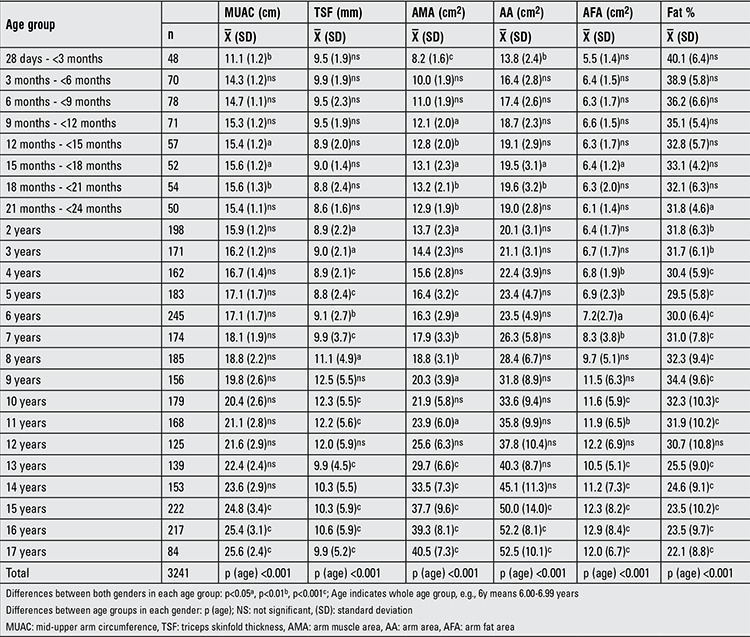
Sample size and mean ± SD values per age group for six anthropometric indices (boys)

**Table 1b:**
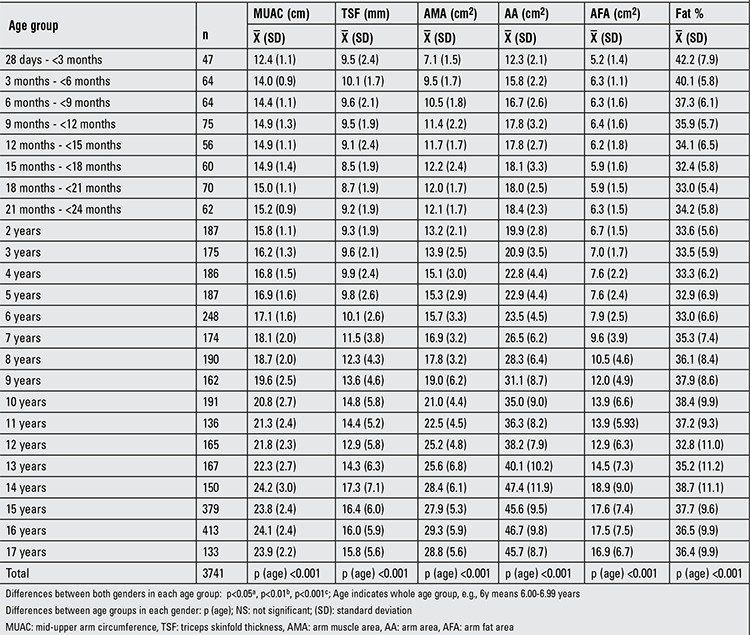
Sample size and mean ± SD values per age group for six anthropometric indices (girls)

**Table 2:**
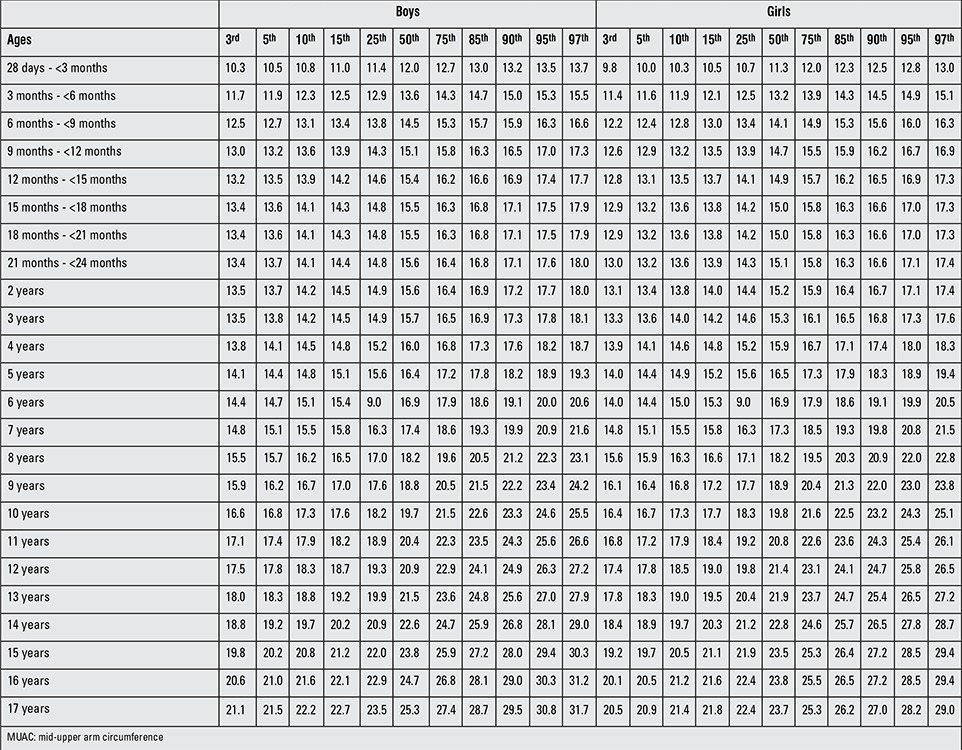
Smoothed age- and gender-specific MUAC percentile values (cm) for Turkish children and adolescents

**Table 3:**
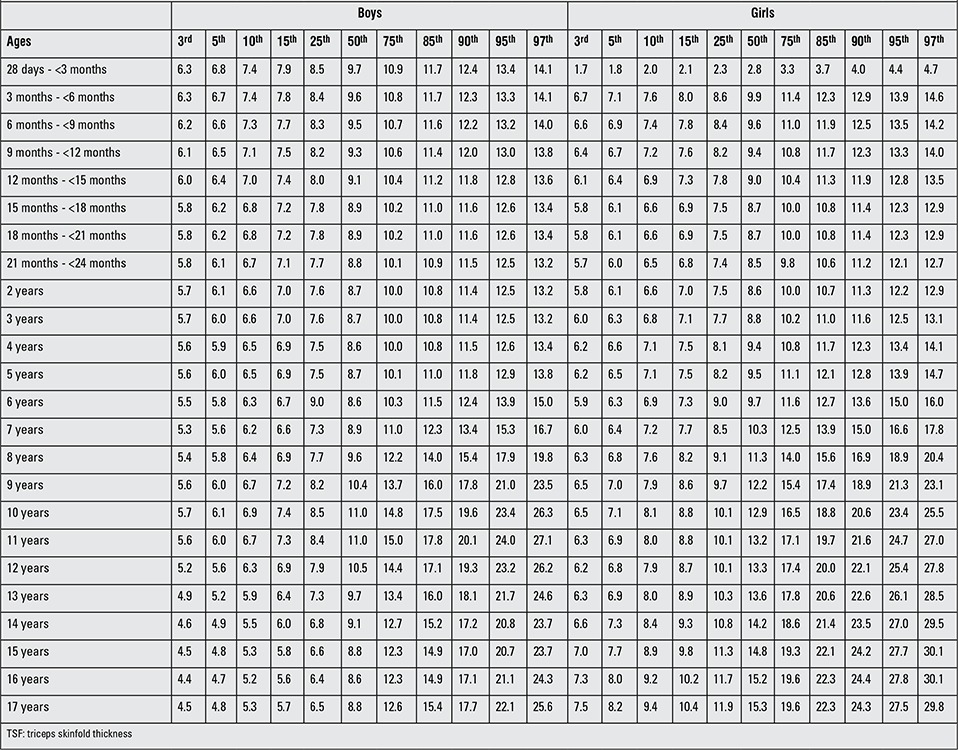
Smoothed age- and gender-specific TSF percentile values (mm) for Turkish children and adolescents

**Table 4:**
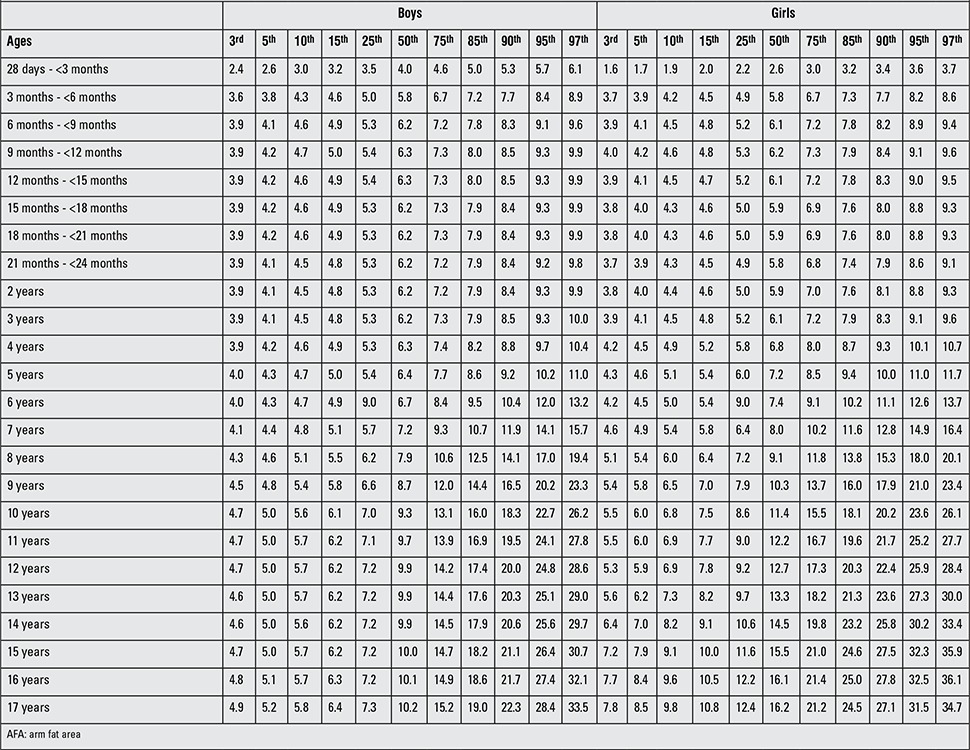
Smoothed age- and gender-specific AFA percentile values (cm2) for Turkish children and adolescents

**Table 5:**
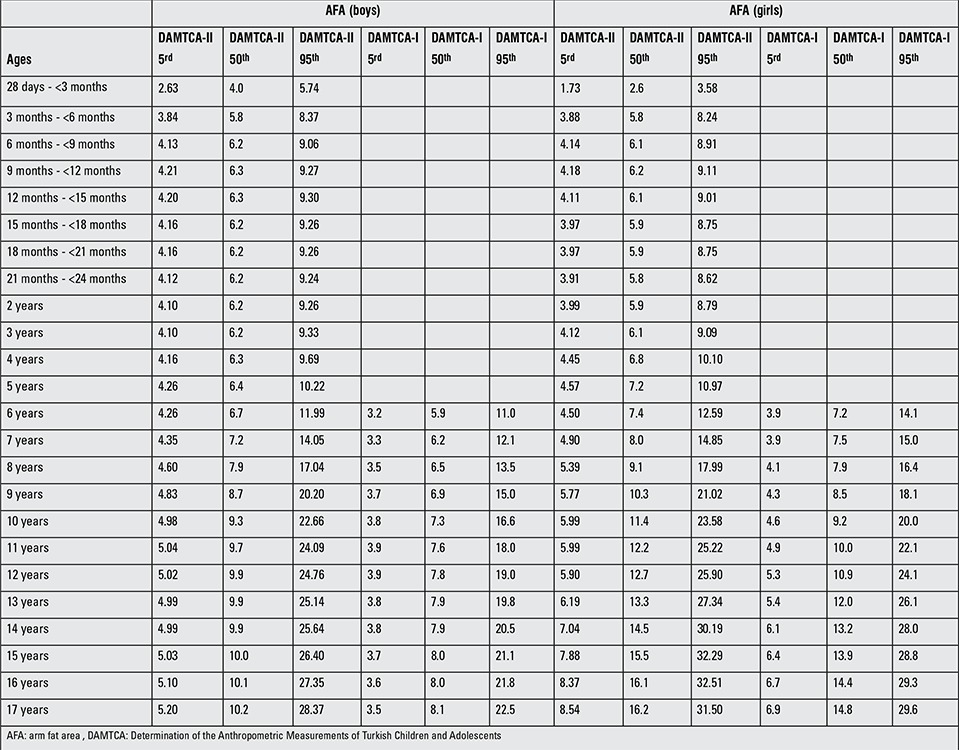
AFA comparisons of DAMTCA-II with DAMTCA-I for 5th-50th-95th percentiles

**Table 6:**
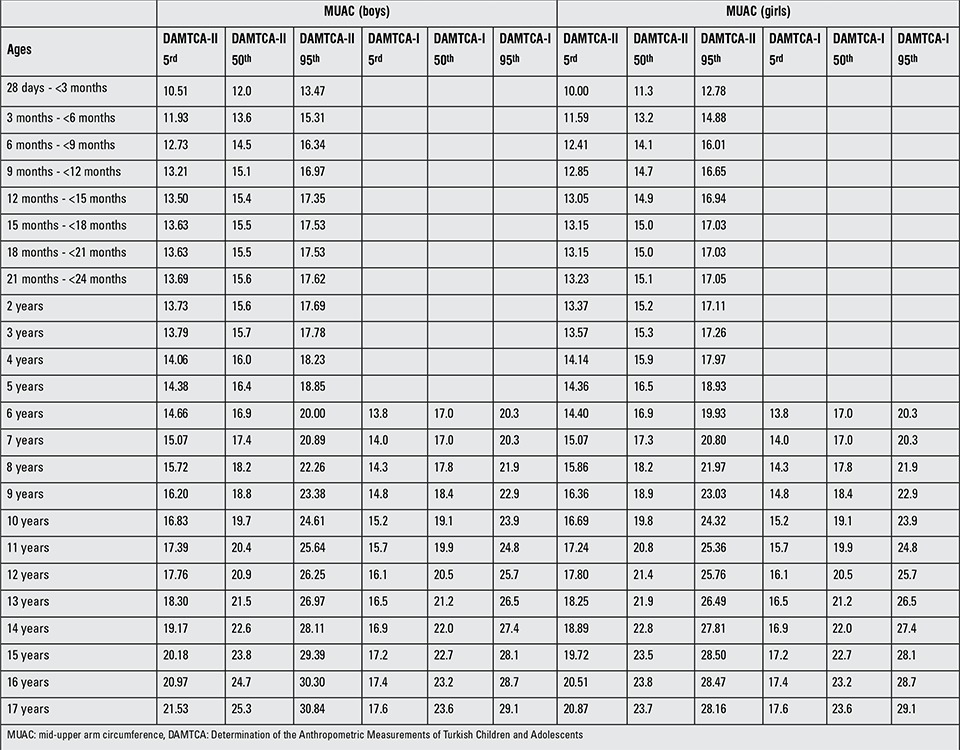
MUAC comparisons of DAMTCA-II with DAMTCA-I for 5th-50th-95th percentiles

**Figure 1a:**
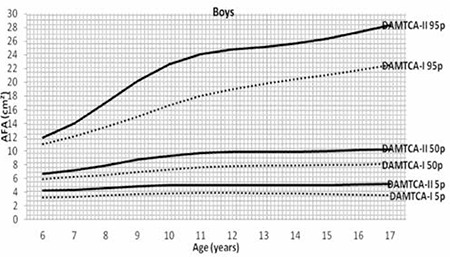
Comparison of arm fat area (AFA) 5th-50th-95th percentiles of Determination of the Anthropometric Measurements of Turkish Children and Adolescents (DAMTCA-I) and DAMTCA-II studies for boys

**Figure 1b:**
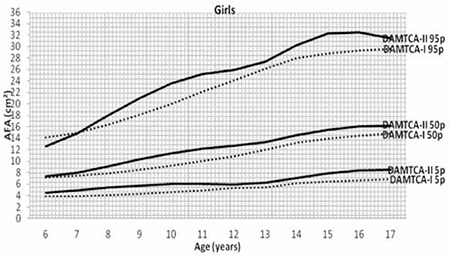
Comparison of arm fat area (AFA) 5th-50th-95th percentiles of Determination of the Anthropometric Measurements of Turkish Children and Adolescents (DAMTCA-I) and DAMTCA-II studies for girls

**Figure 2a:**
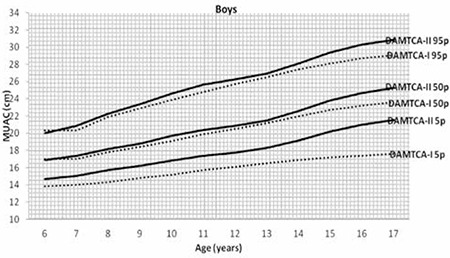
Comparison of mid-upper arm circumference (MUAC) 5th-50th-95th percentiles of Determination of the Anthropometric Measurements of Turkish Children and Adolescents (DAMTCA-I) and DAMTCA-II studies for boys

**Figure 2b:**
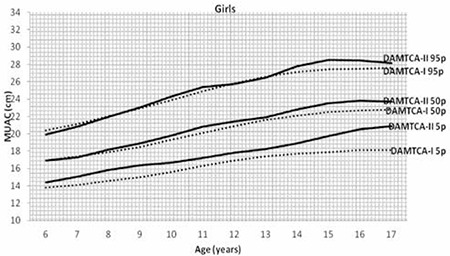
Comparison of mid-upper arm circumference (MUAC) 5th-50th-95th percentiles of Determination of the Anthropometric Measurements of Turkish Children and Adolescents (DAMTCA-I) and DAMTCA-II studies for girls
